# A semi-automated method for counting fluorescent malaria oocysts increases the throughput of transmission blocking studies

**DOI:** 10.1186/1475-2875-9-35

**Published:** 2010-01-29

**Authors:** Michael J Delves, Robert E Sinden

**Affiliations:** 1 Division of Cell and Molecular Biology, Imperial College London, South Kensington, London, SW7 2AZ, UK

## Abstract

**Background:**

Malaria transmission is now recognized as a key target for intervention. Evaluation of the *Plasmodium *oocyst burden in the midguts of *Anopheles spp*. is important for many of assays investigating transmission. However, current assays are very time-consuming, manually demanding and patently subject to observer-observer variation.

**Methods:**

This report presents the development of a method to rapidly, accurately and consistently determine oocyst burdens on mosquito midguts using GFP-expressing *Plasmodium berghei *and a custom-written macro for ImageJ. The counting macro was optimized and found to be fit-for-purpose by performing gametocyte membrane feeds with parasite infected blood. Dissected midguts were counted both manually and using the automated macro, then compared. The optimized settings for the macro were then validated by using it to determine the transmission blocking efficacies of two anti-malarial compounds - dehydroepiandrosterone sulphate and lumefantrine, in comparison to manually determined analysis of the same experiment.

**Results:**

Concurrence of manual and macro counts was very high (R^2 ^= 0.973) and reproducible. Estimated transmission blocking efficacies between manual and automated analysis were highly concordant, indicating that dehydroepiandrosterone sulphate has little or no transmission blocking potential, whilst lumefantrine strongly inhibits sporogony.

**Conclusion:**

Recognizing a potential five-fold increase in throughput, the resulting reduction in personnel costs, and the absence of inter-operator/laboratory variation possible with this approach, this counting macro may be a benefit to the malaria community.

## Background

*Plasmodium*, the causative agent of malaria has a complex life cycle, requiring both vertebrate and mosquito hosts. Merozoites invade mammalian red blood cells (RBCs), wherein they replicate producing schizonts, which rupture the RBCs releasing daughter merozoites that are then free to invade other RBCs. With each such asexual cycle, a small subset of merozoites is committed to form male or female gametocytes, the sexual stages of the parasite that are uniquely infectious to mosquitoes [1]. When a mosquito feeds on blood of an infected host, a drop in temperature and the presence of a specific mosquito-derived factor, xanthurenic acid (XA) in the insect's stomach, within seconds activate gametocytes to form either male of female gametes [2]. Following fertilization in the mosquito midgut, each zygote generates, ~24 hours later, a motile ookinete that glides through the digesting blood meal to the midgut wall. The ookinete passes through the midgut epithelial cell, reaching the basal lamina and transforms into the oocyst that grows and develops over 10-21 days, producing thousands of sporozoites. When the oocysts burst, sporozoites are released into the mosquito haemolymph where they migrate to the salivary glands and ducts, ready to infect another mammalian host [3].

The 'gold standard' assay for measuring factors affecting *Plasmodium *transmission to the mosquito (gametocyte to oocyst transition) is to feed groups of mosquitoes on parasite-infected hosts or infected blood in artificial membrane feeders (the standard membrane feed assay - SMFA), and then count the number of oocysts that develop on the mosquito midgut [4]. This type of assay has been widely used in various forms throughout the literature to assess transmission-blocking compounds [5,6], transmission-blocking vaccines [7-9], mosquito immune responses [10] and to phenotype transgenic parasites [11,12]. Whilst it is an extremely powerful assay, it is very slow, laborious, and subject to significant observer-observer variation (as observed over 30 years of study, RES, personal observation). Presented here, is a semi-automated method to greatly increase the throughput of oocyst counting using GFP-expressing parasites [13], allowing at least a five-fold increase in throughput, repeatable and consistent inter-operator accuracies, thus enabling the larger-scale screening of transmission blocking interventions to become a more realistic prospect.

## Methods

All work involving laboratory animals was performed in accordance with the EU regulations 'EU Directive 86/609/EEC' and within the regulations of the United Kingdom Animals (Scientific Procedures) Act 1986.

### Parasite maintenance

Female TO mice were treated with phenylhydrazine three days prior to infection by i.p inoculation with *Plasmodium berghei *constitutively expressing GFP (PbGFPCON - derived from the ANKA line) [13]. To ensure reproducibility and high infectivity, the parasites had been passaged between mice no more than eight times since previously passing through mosquitoes. After four days of infection, exflagellation was tested by spotting tail blood into ookinete medium on slides for 15 mins at 20°C. If >5 exflagellation centres were observed in each of five random fields of view (×40 objective) in which the blood cells form an even monolayer, then the mouse was exsanguinated and the infected blood used in the SMFA.

### Compounds

Two anti-malarial compounds - dehydroepiandrosterone sulphate (DHEA-S) and lumefantrine (LUM), and a known inhibitor of microgametogenesis and infection (positive control) - cycloheximide (CH) [14], were selected for evaluation of the counting macro. DHEA-S and CH were dissolved in DMSO and LUM was dissolved in dimethylformamide (DMF). All were stored under dry N_2 _gas as 30 mM stock solutions. Compounds and solvents were provided blind to the experimenter and only un-blinded when all experiments were complete. All compounds were used in the transmission-blocking assay with no more than a 0.1% final solvent concentration. 0.1% DMSO or DMF were used as 'solvent controls'.

### Transmission blocking assay

For each of the three replicate experiments, three infected mice (see above) were used per assay. They were anaesthetized, before being rapidly bled by cardiac puncture and their blood pooled. 500 μl of infected blood was mixed with each compound to be tested, or DMSO/DMF as a control, to yield a 10 μM final concentration of compound. The infected, drug-treated blood was then injected into a membrane feeder pre-warmed to 39°C and offered to overnight-starved *Anopheles stephensi *(strain SDA 500) mosquitoes in groups of ~80, which were then allowed to feed on the blood for 30 min [4]. The next day, mosquitoes that had not fed were removed and the remaining mosquitoes maintained at 19°C and 80%RH, being fed on a fructose/p-aminobenzoic acid (PABA) solution [4] which was replenished every 2-3 days. At 7-9 days post feeding, mosquito midguts were dissected into PBS and the pooled material fixed in PBS containing 4% paraformaldehyde (PFA) for 30 min at room temperature. The guts were then washed with PBS and stored in PBS in the dark at 4°C for no more than 3 days before being counted.

### Imaging

Groups of 20 fixed guts were mounted on glass slides containing a small amount of Vectashield mountant and a coverslip applied. To facilitate imaging, guts were then flattened by gently withdrawing some fluid from the preparation. The slides were observed with a Leica DMR microscope at ×5 magnification using a Leica HCX PL FLUOTAR 5×/0.15 lens (to give maximum optical depth of field combined with adequate spatial resolution) and guts imaged with a Zeiss AxiocamHR camera controlled by AxioVision v4.7.2 software. Images from the same set of feeds were all taken at a resolution of 1388 × 1040 pixels with the same exposure setting and guts were all imaged on the same day under the same conditions. This was done to ensure that there was minimal variation in fluorescence intensity between guts due to the degradation of GFP within the oocysts over the time they were stored.

## Results and Discussion

### Algorithm

**Figure 1:**
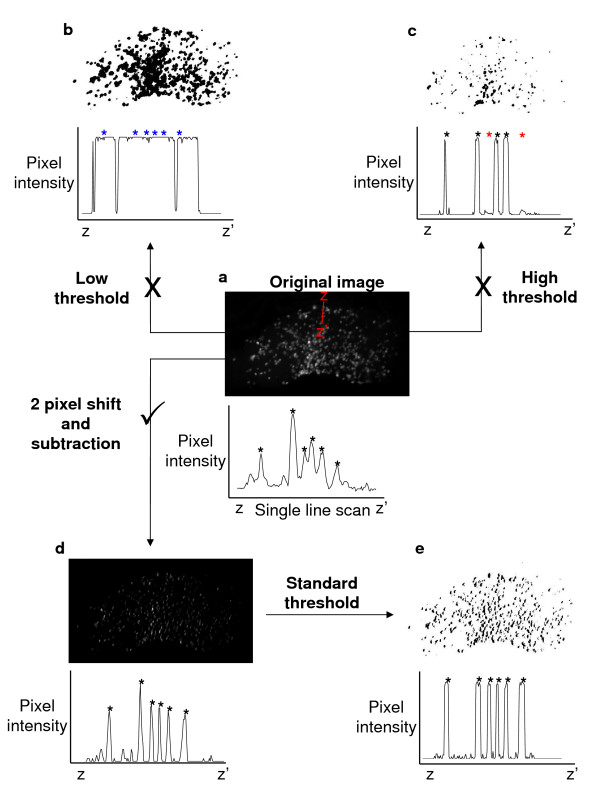
**Methods of digital processing to identify oocysts on infected mosquito midguts**. (a) A microscope image of a mosquito midgut infected with GFP-expressing parasites can be separated into two distinct components: 1. Small, brightly fluorescent oocysts; 2. Faint, non-uniform autofluorescent midgut tissue. The graph underneath denotes the pixel intensity along the line Z to Z'. Black asterisks correspond to oocysts identified manually. It is not possible to accurately separate out the oocyst component of the image by thresholding alone. (b) With a low threshold level, midgut tissue remains in the image, thus obscuring some oocysts (blue asterisks). (c) A high threshold level eliminates the background midgut tissue fluorescence, but some fainter fluorescence oocysts are lost also (red asterisks). (d) Applying a 2 pixel horizontal shift (equivalent to ~1 μm of the specimen) to a copy of the image, followed by a subtraction of the copy from the original image cancels out most of the midgut background autofluorescence whilst preserving the oocysts. (e) With background fluorescence eliminated, a standard threshold level can be applied to all gut images that accurately separates the oocysts from midgut tissue.

The acquired images of the infected mosquito midguts can be broken down into two components: 1. Oocysts that are small (~7-15 pixels/14-30 μm diameter), round and show high GFP fluorescence; 2. Midgut tissue that is larger than the oocysts (~600 × 400 pixels/1200 × 800 μm), and has fainter variable and non-uniform yellow-green autofluorescence. Even though oocysts are brighter in greyscale than the surrounding midgut tissue, it is impossible to reliably separate them by simply thresholding the image (Figure 1). A low threshold level will leave traces of the gut in the image and thus obscure the oocysts (Figure 1b), whereas a high threshold level despite giving a cleaner image, does so at the cost of losing oocysts with fainter fluorescence (Figure 1c). Also, as midgut background fluorescence varies between guts, it is impossible to set a standard threshold level that will apply to all images to give consistency in analysis.

An automated oocyst counter must therefore be able to process each image in exactly the same manner to give reproducible data, therefore, background fluorescence must be standardized before an accurate counting analysis can take place. Experimentally this is very difficult to achieve and so a macro for ImageJ was developed that can take any midgut image and process it with a simple set of standard image transformations that remove the background tissue fluorescence whilst preserving the oocyst component of the image (Figures 1d and 1e).

The background fluorescence for each image is minimized by taking a copy of the image, displacing it horizontally by 2 pixels (equivalent to ~1 μm of the specimen), and then subtracting it from the original image. Subtracting a copy of the image slightly out of phase with the original causes large areas of relatively uniform and weak fluorescence intensity to be cancelled out (midgut tissue). Small areas (~7-15 pixels diameter) of bright fluorescence (oocysts), whilst diminishing in size and intensity remain distinct (Figure 1d). A standard threshold can then be applied to all images such that they highlight only the oocysts (Figure 1e). The standard threshold level that correctly identifies the oocysts is determined by visually evaluating three representative images from the dataset, and then is applied to all images in the batch of images for a particular group of feeds. The macro then uses the particle counter function built-in to ImageJ to count the oocysts it has identified. The counts generated are then automatically tabulated, and can be imported into MS Excel or similar programs for analysis.

### Testing - Calibration and accuracy of counting macro

The ImageJ particle counter function used by the counting macro allows the user to specify the minimum particle size counted. ImageJ defines a particle as an object of undefined shape with an area measured in pixels squared (pixels^2^). For example, objects of 2 × 2 pixels and 1 × 4 pixels in size are both classified as being 2 pixels^2^. This value was hypothesized to be critical for the accuracy of counting. To test this, gut images from three gametocyte membrane feeds (n = 45, 49 and 51 mosquitoes) were counted by the macro using different minimum particle sizes. To enable the precision of the macro counts to be evaluated, the same midgut images were then recounted and recorded manually by an experienced trained observer. The observer recorded each identified oocyst individually and used their own judgement to discriminate between overlapping oocysts.

**Figure 2:**
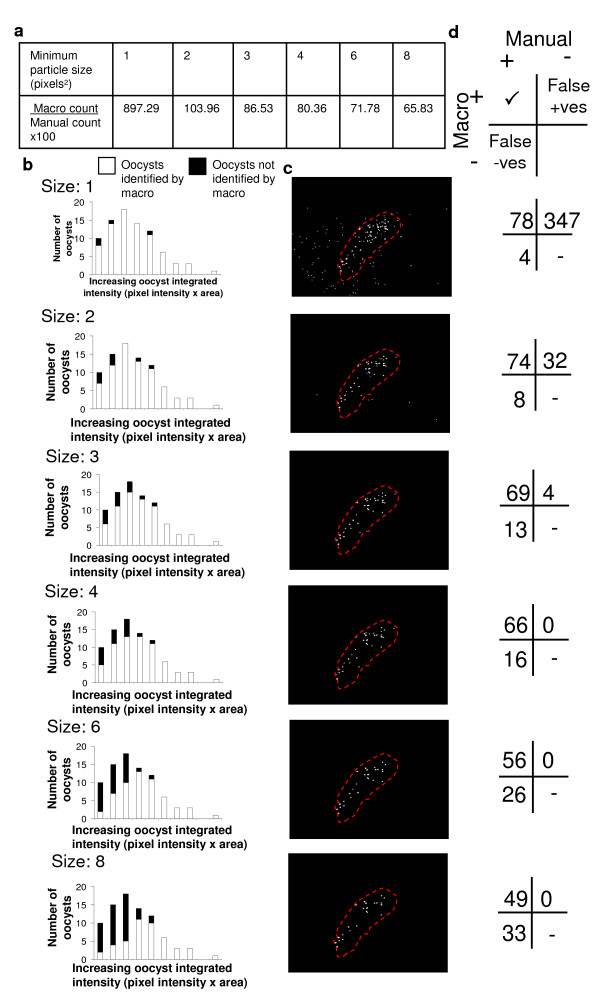
**Investigating the effects of different minimum particle sizes on the counting accuracy of the macro**. It is possible to specify the minimum particle size counted by the ImageJ particle counter used to identify oocysts in the macro (measured in pixels^2^). The effect of this on the ability of the macro to accurately count oocysts was investigated. Three gametocyte membrane feeds were carried out and the parasite-infected guts were imaged at day 7-9 after feeding (n = 45-51 mosquitoes). (a) The images were analysed by the macro using different minimum particle sizes and the reported mean oocyst intensity compared to that determined by manual observation of the images. A minimum particle size of 1 or 2 pixels^2 ^over-counted the mean number of oocysts present per gut. At 3 to 8 pixels^2^, the macro progressively under-counted the number of oocysts present. (b) The integrated fluorescence intensity (pixel intensity × oocyst area) of individual oocysts was measured for three random images from the feeds and it was recorded whether the counting macro had detected or missed each them at different minimum particle sizes. The macro was found to be less likely to identify oocysts with low integrated fluorescence intensity and that this effect increased with increasing minimum particle size. (c) Representative image of an oocyst-infected mosquito midgut (red dashed line) showing the particles identified as oocysts at different minimum particle sizes (white dots). At low minimum particle sizes, many false positives are generated. (d) A minimum particle size of 3 pixels^2 ^was determined to give the counting macro the best balance between false positives to false negatives.

It was found that by increasing the minimum particle size the counter detects from 1 to 8 pixels^2^, the number of particles identified by the macro as oocysts decreased - with 1 pixel^2 ^notably over-counting (by 897.29%) and 8 pixels^2 ^under-counting (65.83% of manual count) (Figure 2a). To investigate this effect, three images were randomly selected from the experiments and the fluorescence intensity of the individual oocysts present in the guts (n = 81 oocysts) measured and related to whether that particular oocyst was counted or not for different minimum particle sizes (Figure 2b). The macro was most insensitive to identifying oocysts that had a low integrated fluorescent intensity (pixel intensity × area). With increasing minimum particle size, the population of oocysts with low integrated intensity that were not identified rose, and also brighter (higher integrated intensity) oocysts began to be missed (Figures 2b and 2d). The over-count of oocysts produced by a minimum particle size of 1 or 2 pixels^2 ^(Figure 2a) was found to be due to false identification of random pixel noise that was smaller in size than the oocysts (Figures 2c and 2d).

The effect of particle size on calculating the prevalence of infection was also tested by imaging 100 uninfected guts, and then counting them using the macro set at the different minimum particle sizes (Table 1). Due to high false-positive rates a minimum particle size of 1 or 2 pixels^2 ^gave a prevalence of 100%. At 3 pixels^2 ^this dropped to 9% and by 6 pixels^2 ^had reduced to 2%. The 2 images that the macro incorrectly identified at this size contained either abnormally high background fluorescence in the midgut tissue or contained a single fluorescent artefact that was the same size and shape as an oocyst.

**Table 1:** Comparison of the effect of minimum particle size on the calculated prevalence of uninfected guts

Minimum particle size (pixels^2^)	1	2	3	4	6	8
Prevalence of 100 uninfected guts as reported by the macro (%)	100	100	9	7	2	2

Altering the minimum particle size used to identify oocysts by the counting macro affected the calculated prevalence of infection. 100 images of uninfected guts were counted by the macro using different minimum particle sizes. Due to many false positives, a minimum particle size of 1 to 2 pixels^2 ^gave 100% prevalence. This dropped to 9% for 3 pixels^2 ^and 2% by 6 pixels^2^.

As no setting identified oocysts with 100% accuracy, a minimum particle size of 3 pixels^2 ^(recognizing objects in the processed image containing 9 pixels or greater) was selected for use in further experiments with this material as it most closely balanced out the false positives and false negatives produced by the macro (Figure 2d).

**Figure 3:**
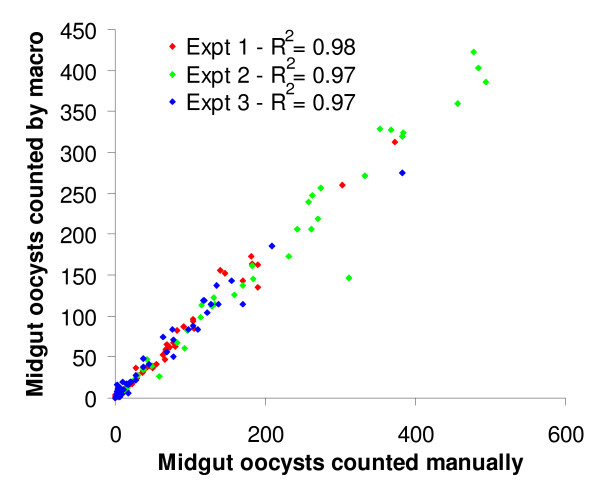
**Manual counts plotted against automated counts for three replicate feeds**. The oocyst counts for individual guts from the triplicate gametocyte membrane feeds both counted manually and by the macro were plotted on a scatter plot. Trendlines were fitted to the data (not shown) showing that the counts by the macro in all three experiments were highly consistent (Mean R^2 ^= 0.973).

To check for reproducibility, the oocyst counts for individual infected midgut images from the three test gametocyte feeds analysed with a minimum particle size of 3 pixels^2 ^were plotted on a graph against the corresponding manually determined oocyst count (Figure 3). Trendlines were fitted to the data (not shown) that gave R^2 ^values of 0.98, 0.97 and 0.97 respectively (Figure 3), suggesting that although at this setting the macro under-counts the number of oocysts present by 13.47% (Figure 2a, 100% minus 86.53), most importantly it is invariant in the manner that it treats each image.

### Implementation - Oocyst counting macro closely correlates with manual count for two anti-malarial compounds

10 μM concentrations of CH, DHEA-S, and LUM, and DMSO or DMF-only carrier controls were added to parasite infected blood and fed to mosquitoes (n = 28-58) in triplicate experiments. At day 7-9 the guts were dissected, imaged and then counted both by the macro at a minimum particle size of 3 pixels^2 ^and manually. As the mean oocyst intensities of the solvent control varied between the three replicates, (for DMSO, between 71.51 and 180.37 oocysts per gut by manual count), feeds were analysed by expressing the oocyst intensity for each individual replicate as a percentage of its respective solvent control, and then taking the mean of the three replicates (Table 2). This was performed with the oocyst intensities generated for either the manual or automated counting methods and then compared. Both counting methods firmly agreed that DHEA-S was a very weak inhibitor of sporogony (Manual = 17.79% reduction in oocyst intensity; Automated = 27.41% reduction in oocyst intensity), whilst LUM (Manual = 89.15% reduction; Automated = 84.75% reduction) strongly inhibited sporogony. The prevalence of infection as determined by the macro count agreed well with the manually determined prevalence (Table 2), with LUM showing a statistically significant decrease in prevalence (P =< 0.004 unpaired Student's T-Test). DHEA-S has been shown to exhibit anti-malarial activity by inducing phagocytosis of asexual ring stage parasites [15]. The data presented here suggests that it exerts little or no effect on mosquito stage parasites (Table 2). LUM has been reported to be schizonticidal [16], most likely exerting its effects through interacting with the heme detoxification pathway [17] and is most commonly used today in combination with artemether (Coartem^®^) for treating malaria [18]. Interestingly, it exhibits a strong inhibition of sporogony in *P. berghei *(Table 2). The 10 μM concentration we have tested is comparable to serum levels detected in malaria patients treated with LUM [19]. As a consequence, it would be prudent to investigate whether LUM affects *P. falciparum *in a similar manner.

**Table 2:** Comparison of the transmission blocking potential of two anti-malarials determined by manual and automated counts

Manual count				
	**Intensity as a % of ** **solvent control (SEM)**	**Mean prevalence ** **% (SEM)**	**Reduction in intensity ** **% (SEM)**	**Reduction in prevalence ** **% (SEM)**

DMSO	100 (0)	91.05 (2.95)	0 (0)	0 (0)

DMF	100 (0)	96.90 (0.36)	0 (0)	0 (0)

CH	0 (0)	0 (0)	100 (0)	100 (0)

DHEA-S	82.21 (12.84)	72.78 (11.53)	17.79 (12.84)	10.18 (4.73)

LUM	10.85 (3.35)	69.45 (5.66)	89.15 (3.35)	28.3 (6.01)

**Automated count**				

	**Mean oocyst intensity as ** **a % of solvent control (SEM)**	**Mean prevalence ** **% (SEM)**	**Reduction in intensity ** **% (SEM)**	**Reduction in ** **prevalence % (SEM)**

DMSO	100 (0)	95.85 (4.87)	0 (0)	0 (0)

DMF	100 (0)	94.60 (1.51)	0 (0)	0 (0)

CH	0.20 (0.13)	5.61 (0.68)	99.80 (0.13)	94.32 (0.62)

DHEA-S	72.59 (3.57)	89.26 (5.25)	27.41 (3.57)	4.37 (4.06)

LUM	15.25 (3.98)	71.95 (8.12)	84.75 (3.98)	24.18 (8.12)

The automated counting macro produced oocyst intensity and prevalence figures closely matching those determined by a manual count. The two anti-malarial compounds and controls were fed to mosquitoes (n = 28-58) in triplicate experiments. 7-9 days after feeding, the midguts were dissected and imaged before being counted both manually and by the counting macro. The mean oocyst intensity is expressed as a percentage of the solvent control, and the reduction in intensity is expressed as 100% minus the mean oocyst intensity. The mean prevalence is expressed as the mean percentage of the number of infected mosquitoes for each replicate, and reduction in prevalence is calculated using the following formula: (individual replicate prevalence/corresponding control prevalence) ×100, then taking the mean of the three replicates and subtracting it from 100%. Calculated standard error of the mean in brackets. By manual count of the parasite-infected midguts, LUM and CH showed a marked inhibition of sporogony. DHEA-S however showed minimal effect on sporogony. The automated count by the macro concurred well and showed no significant deviation from the manually determined counts. The prevalence of infection recorded by the counting macro also closely matched the manually determined values.

By manual count, the earlier observations of Toyé et al [14] were confirmed in that the protein synthesis inhibitor CH totally inhibited parasite development, and all guts were free of oocysts. The counting macro nonetheless returned a mean oocyst number of 0.20% for CH compared to the DMSO control, and mean prevalence of 5.61%. When specifically reviewing these images, falsely reported oocysts were identified as containing oocyst-sized fluorescent artefacts. This may be controlled experimentally by imaging and counting a series of uninfected guts at the same time as the experimental guts and taking the calculated prevalence in the uninfected controls as the 0% prevalence baseline for that experiment.

Under the conditions described in this report, researchers were able to perform 20 experiments per week on a sustained basis using the macro. This compares very favourably with manual counting, which permits only four experiments per week to be completed on a sustained basis. The counting macro, therefore, as well as saving time, also reduces the cost of performing SMFAs and increases the reproducibility of their analysis.

## Conclusions

Assays for transmission blocking vaccines/drugs and phenotypic analyses are vital for the study of *Plasmodium *development in the mosquito but remain very low throughput due to the complexity of the experimental set up, and the laborious man-hours of repetitive observations needed for analysis. The oocyst counting macro greatly reduces the time and labour needed to analyse such assays, allowing for greater throughput and massively increased productivity. Despite slightly under-estimating the oocyst intensity and over-estimating the prevalence, it gives highly consistent (R^2 ^= 0.97-0.98) and unbiased counts, well within the range of operator-operator variability and also never suffers from counting fatigue. Whilst automated counting systems have been developed for determining blood-stage parasite burdens [20,21], currently there are no other published automated counting systems for evaluating midgut oocyst burden and so it is anticipated that it will be a valuable tool.

Although the macro settings were optimized to detect *P. berghei *day 7-9 oocysts, recently a *P. falciparum *transgenic line constitutively expressing GFP throughout its life cycle has been generated (Talman AM, Blagborough AM, Sinden RE: A Plasmodium falciparum strain expressing GFP throughout the parasite's life cycle, PLoS One, in press). Initial testing has shown that the counting macro is applicable to this parasite line also. It is anticipated that the counting macro will be easily adaptable to any technique that renders oocysts a sufficiently contrasting colour to midgut tissue. The counting macro maximizes the throughput of transmission blocking mosquito-based assays in their current format. There still remain bottlenecks to further improvements in throughput, such as dissecting the mosquitoes and faster imaging. Automated microscopy techniques may reduce the latter. The former would require a more drastic reworking of current assay procedures to perhaps include reporter parasites that can be detected either *in situ *in the mosquito, or from crude mosquito extract (such as luciferase reporter genes). These approaches however may not give as useful information as they would assess parasite biomass, which certainly does not correlate directly with parasite number.

This report demonstrates and validates the utility of the macro in accurately determining and reporting the transmission blocking activity of two anti-malarial compounds. With the growing recognition that blocking parasite transmission to the mosquito is likely to play an important role in the eradication of malaria [22,23], this macro is offered as a free download to the research community [24] under a Creative Commons Attribution-Non-Commercial-Share Alike licence 2.0.

## Competing interests

The authors declare that they have no competing interests.

## Authors' contributions

MJD conceived and designed the algorithm, and performed the experiments described here. MJD and RES participated in drafting the manuscript. All authors read and approved the final manuscript.
